# Sensory Changes Following the Lateral Nasal Wall Surgery: A Systematic Review

**DOI:** 10.7759/cureus.75628

**Published:** 2024-12-13

**Authors:** André Sousa-Machado, Rafaela Fernandes, Mariana Cascao, Patricia Sousa, Antonio Castanheira

**Affiliations:** 1 Otolaryngology, Local Health Unit of Trás-os-Montes and Alto Douro (ULSTMAD), Vila Real, PRT; 2 Medical Education and Simulation, Faculty of Health Sciences, University of Beira Interior, Covilhã, PRT

**Keywords:** lateral nasal wall surgery, meta-analysis, nose surgery, sensory changes, sensory disturbances

## Abstract

The objective of this study was to analyze the sensory changes reported by patients after lateral nasal wall surgery and to assess the prevalence and severity of sensory disturbances, the factors that influence their occurrence, and their impact on patients’ quality of life. The methodology adopted in this study was PRISMA (Preferred Reporting Items for Systematic Reviews and Meta-Analysis), as well as the PICO (Population, Intervention, Comparison, Outcomes) strategy, which assisted in the development of the study’s objectives. Based on the inclusion and exclusion criteria, among all the articles retrieved in the searched databases (PubMed and Google Scholar), we selected 15 articles, considered to be pertinent and relevant to the present investigation. The results demonstrated that there are several sensory disturbances in patients, highlighting the importance of tailoring the best surgical technique to tackle the problem and, consequently, improve patients’ quality of life. Overall, all 15 articles demonstrated important outcomes for patients’ health, contributing to their recovery and changing their initial status. No consensus emerges regarding the most suitable surgical technique, as has been established by previous literature. In conclusion, the revision showed that sensory changes reported by patients are congruent with the previously identified sensory disturbances. Surgical procedures must be tailored by surgeons precisely to treat the previously identified sensory disturbances, thus contributing to the increase in patients’ quality of life.

## Introduction and background

Nasal obstruction significantly impacts patients' quality of life, with hypertrophy of the inferior turbinate being the most prevalent cause of nasal airway obstruction. This condition often arises from rhinosinusitis, allergic rhinitis, or non-allergic rhinitis [1-3]. When pharmacological interventions fail, surgical options targeting the inferior turbinate may be considered. These include turbinoplasty, partial or total turbinectomy, laser cautery, electrocautery, and innovative methods such as ultrasound turbinate reduction, microdebrider submucous reduction, and radiofrequency techniques [4]. Although short-term improvements have been observed following these procedures [5], the long-term outcomes are generally unsatisfactory, and no clear consensus exists regarding the optimal surgical approach with minimal patient morbidity [6].

The inferior turbinate plays a critical role in regulating nasal airflow and resistance, being intricately linked to the nasal valve area - the narrowest segment of the nasal airway. Effective management of dysfunctions in this area requires a deep understanding of both normal anatomy and the pathophysiology underlying common abnormalities [7]. Causes of nasal valve dysfunction are classified as either primary or secondary. Primary causes, which are relatively rare, include conditions such as a narrow pyriform aperture, weakened or concave upper and lower lateral cartilages, a lack of overlap between these structures, or prolapse of the upper lateral cartilage into the airway [8]. In contrast, secondary causes are more frequent and include septal deviation, turbinate hypertrophy, scarring, and over-resection of the lower lateral cartilage's caudal border [9]. Additional minor contributors may involve reduced strength in the levator labii superioris alaeque nasi muscle [10].

Pyriform turbinoplasty represents one surgical option for improving nasal airflow, targeting the bony structure to reshape the area without removing the mucosa of the inferior turbinate. This procedure involves a submucosal resection of the frontal maxillary process and part of the lacrimal bone [11-13]. Beyond this, various other techniques designed to enhance airflow continue to emerge in the literature. These methods commonly aim to expand the nasal valve area, either by minimizing the narrowing of the valve angle during inspiration, or by increasing the valve angle itself. Often, these procedures are combined with lateralization or reinforcement of the lateral nasal wall. Despite these advances, no definitive agreement exists regarding the most effective surgical strategy to address nasal valve insufficiency [14]. Ideally, surgical approaches should be precisely adapted to the specific site and nature of the deformity or abnormality affecting the internal or external nasal valves [15].

This study aims to explore two primary objectives: (1) to evaluate the sensory changes experienced by patients following lateral nasal wall surgery, and (2) to examine the prevalence, severity, and contributing factors of sensory disturbances, as well as their impact on patients' quality of life. By conducting this systematic review and analysis of the literature, the goal is to provide an evidence-based, comprehensive understanding of sensory changes associated with lateral nasal wall surgery, and to identify factors influencing these changes and their implications for patients' well-being.

## Review

This article is a systematic review and analysis study based on the Preferred Reporting Items for Systematic Reviews and Meta-Analysis (PRISMA) methodology. As such, the process of selection and identification of the articles was carried out in August 2023 by an independent researcher.

The research question of the present study resulted from the PICO (Population, Intervention, Comparison, Outcomes) strategy, which refers to the first stage of the meta-analysis. Essentially, this strategy considers four specific components, more precisely: Patients, Intervention, Comparison, and Outcomes. Therefore, based on these components, the research question comprehends the following factors: PICO strategy - Population: patients who have undergone lateral nasal wall surgery; Intervention: lateral nasal wall surgery (including procedures like pyriform turbinoplasty, lateral nasal wall lateralization, and others); Comparison: sensory changes before and after lateral nasal wall surgery; Outcomes: prevalence and severity of sensory disturbances, factors influencing their occurrence, and impact on patients’ quality of life.

The databases consulted and used in this study were PubMed and Google Scholar, and the descriptors used to conduct the search for articles published in these databases, based on the Boolean operator AND, were: “surgery,” “lateral nasal wall,” “sensory changes,” “lateral nasal wall surgery,” and “sensory disturbances.”

The inclusion criteria defined in this investigation, to guide the selection of articles after searching in the databases, were the following: studies published in peer-reviewed journals; studies that include patients who underwent lateral nasal wall surgery, regardless of the specific surgical technique used; studies that assess sensory changes (e.g., hypoesthesia, paresthesia, altered sensation) following surgery; studies that report sufficient data on the prevalence and severity of sensory disturbances in the postoperative period; and studies that include patients of all age groups and both genders.

Consequently, the established exclusion criteria were based on the following aspects: case reports, editorials, letters, or review articles without original data; studies that do not specifically evaluate sensory changes following lateral nasal wall surgery, with small sample sizes (less than 10 patients) or inadequate data reporting, and with incomplete or inconsistent outcome measures related to sensory changes; and studies that focus solely on specific conditions (e.g., tumors, trauma) that require nasal surgery but are not directly related to the lateral nasal wall.

Results

**Figure 1:**
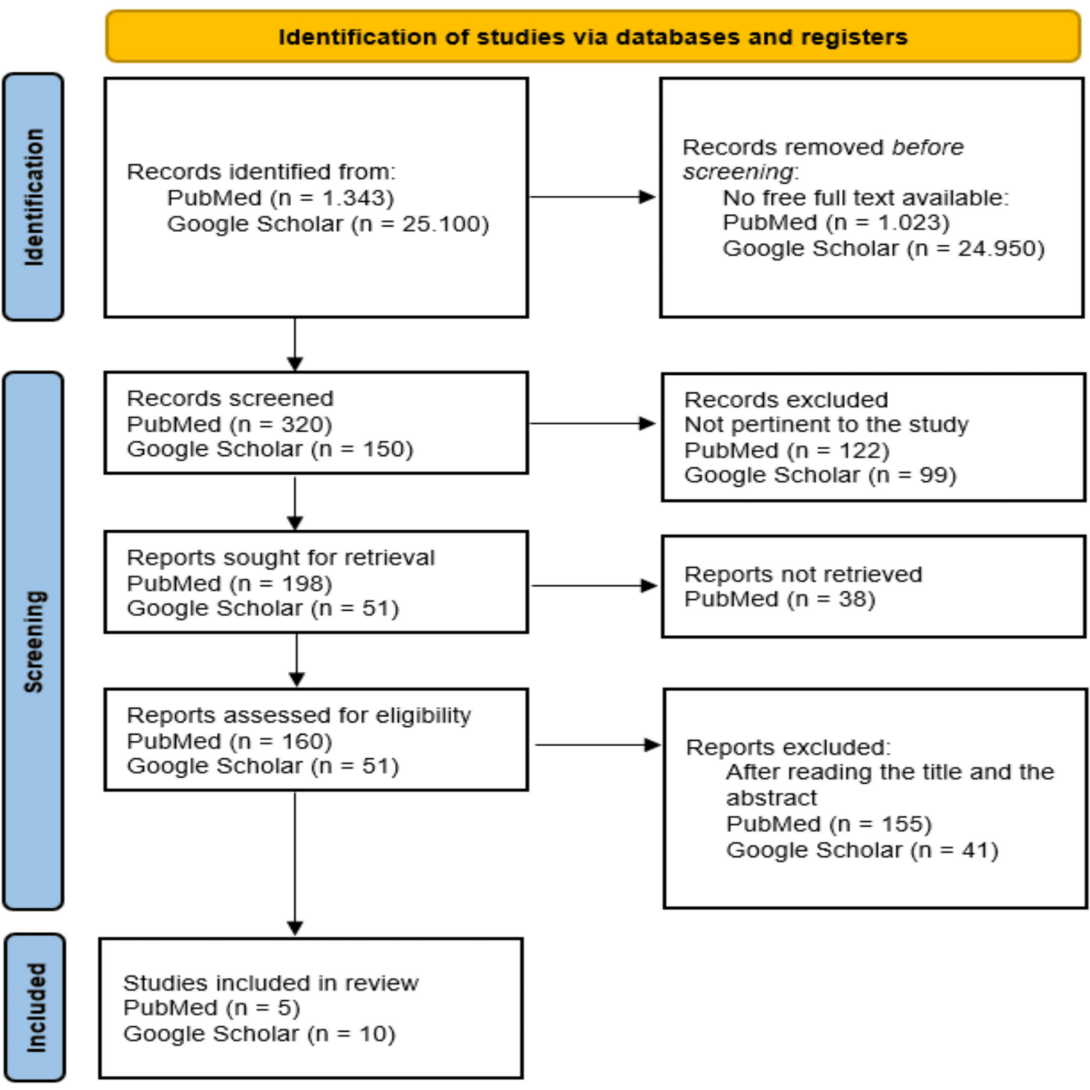
PRISMA flow diagram of the selection process of the articles PRISMA, Preferred Reporting Items for Systematic Reviews and Meta-Analysis

Figure 1 shows the flowchart of the selection process of the articles, based on the recommendations provided by the PRISMA methodology. In sum, 15 studies were selected for this meta-analysis, essentially because they met the inclusion criteria, as well as the PICO strategy, and were pertinent and relevant to fulfilling the study’s objectives.

The 15 selected articles were published between 2000 and 2022, comprising several types of sensory disturbances: (1) chronic rhinosinusitis; (2) odor indiscrimination; (3) poor quality sleep; (4) inflammatory obstruction, impaired olfaction; (5) destruction of normal nasal tissue; (6) loss of smell; (7) sensorineural damage; (8) sinusitis; (9) nasal airway obstruction; (10) severe allergic rhinitis; (11) moderate and severe obstructive sleep apnea; and (12) nerve paraesthesia. Similarly, all studies presented different sample sizes, methods (surgeries and visual imaging), results, and conclusions.

The investigation of nasal airway obstruction across five studies revealed varied yet valuable outcomes, each contributing to the broader understanding of the condition (Table 1). Sullivan et al. [13] observed a significant postoperative increase in mucosal cooling among 10 patients following surgical intervention for nasal obstruction, which correlated positively with patients' self-reported improvement in nasal patency. Kandathil et al. [14] analyzed functional, cosmetic, and combined rhinoplasty cases, recommending that repair of the nasal wall should be considered only in patients demonstrating a positive modified Cottle maneuver. Meanwhile, Stolovitzky et al. [15] examined the outcomes of 101 patients treated with a bioabsorbable implant designed to support the lateral wall, with or without concurrent septoplasty and turbinate reduction. Their findings highlighted a significant reduction in nasal obstruction over a six-month period, underscoring the implant's effectiveness in stabilizing the lateral nasal wall and alleviating obstructive symptoms.

**Table 1:** Techniques and sites to treat nasal valve insufficiency Adapted from André et al. [3]

Site of deformity	Technique	Studies
Apex of internal nasal valve	Spreader grafts (endonasal)	André et al. [5]; Sheen [12]
Bilateral internal nasal valve/upper lateral cartilages and cephalic border of external nasal valve	Butterfly graft	Hage [8]
Lateral wall of internal and/or external nasal valve	Nasal valve suspension	Paniello [16]
Lateral wall of internal and/or external nasal valve	Sub-alar batten grafts	André et al. [4]
Caudal and/or dorsal nasal septum (deformity)	Septal battens or major septal replacement	Aksakal [2]
Caudal and/or dorsal nasal septum (perforation)	Nasal septum perforation repair	Re et al. [17]

Vaezeafshar et al. [18] conducted a comparative study on patients undergoing septorhinoplasty with and without lateral wall repair. Their results demonstrated a significant postoperative decrease in lateral wall insufficiency grades in the group that received repair, aligning their outcomes with those of the control group and emphasizing the importance of tailoring surgical treatment to the specific location of insufficiency. Han et al. [19] evaluated the application of a temperature-controlled radiofrequency device for nasal valve treatment, concluding that it significantly improved patients' quality of life by reducing symptoms such as nasal congestion, headaches, sleep disturbance, daytime sleepiness, and snoring.

The studies examining other sensory disturbances also provided critical insights, all showing positive contributions to patients' quality of life. Turbinate resection was noted as a rehabilitative option that can improve quality of life through nasal augmentation to restore anatomy [20]. Understanding the pathophysiological basis of complications remains essential, particularly when selecting the most suitable surgical techniques [21,22]. Posterior nasal neurectomy, as a minimally invasive procedure, preserves the sphenopalatine artery and effectively reduces postoperative complications (Table 2) [22].

**Table 2:** Summary of the selected articles (author, sample, method, results, sensory disturbances, and conclusions) CT, computed tomography; MRI, magnetic resonance imaging

Author	Sample	Method	Results	Sensory disturbances	Conclusion
Kern [11]	30 patients	Olfactory biopsy at the time of surgery	10 patients demonstrated pathological changes in the olfactory mucosa	Chronic rhinosinusitis	Olfactory deficits in the patients can result from inflammatory changes within the olfactory mucosa in addition to any alteration in airflow to the olfactory cleft
Damm et al. [9]	50 patients	Magnetic resonance imaging of the nasal cavity after olfactometry	Significant correlations between odor thresholds and nasal cavity volumes	Odor indiscrimination	Volume can be modified through resection of the inferior turbinate and/or septoplasty
Vaezeafshar et al. [18]	88 patients	Comparative study on septorhinoplasty with and without lateral wall repair	Postoperative lateral wall insufficiency significantly decreased in the repair group	Nasal obstruction	Localizing lateral wall insufficiency helps tailor surgical treatment
Han et al. [19]	108 patients	Temperature-controlled radiofrequency nasal valve treatment	Improved symptoms over 12 months	Nasal airway obstruction	Significant improvement in nasal congestion, sleep disturbances, and snoring
Chhabra and Houser [7]	1001 patients	Turbinate resection	Prevents the occurrence of empty nose syndrome	Destruction of normal nasal tissue	Turbinate resection with augmentation can restore nasal anatomy and improve quality of life
Udaka et al. [22]	23 patients	Posterior nasal neurectomy	No major complications like nasal bleeding or numbness	Severe allergic rhinitis	Posterior nasal neurectomy is minimally invasive and preserves the sphenopalatine artery
Nguyen et al. [23]	39 patients + 17 controls	Physiologic testing of obstructive sleep apnea and non-snoring controls	Impairment in sensory detection threshold for obstructive sleep apnea vs. control subjects	Poor quality sleep	Sensory function is impaired at multiple upper-airway sites in obstructive sleep apnea
Trotier et al. [24]	34 patients	Location and extension of obstructed clefts (endoscopy, CT scans, MRI)	Sensory deficits equivalent to anosmic patients, with inflammation in the olfactory clefts	Inflammatory obstruction, impaired olfaction	Local inflammatory events in olfactory clefts contribute to sensory deficits unresponsive to corticoids or antibiotics
Sullivan et al. [13]	10 patients	CT scans after nasal obstruction surgery	Heat loss increased significantly postoperatively	Nasal airway obstruction	Postoperative mucosal cooling correlates with better nasal patency
Georgel et al. [25]	27 woodworkers	CT scans of patients with olfactory cleft adenocarcinomas	Adenocarcinoma remodels the olfactory cleft, with loss of nasal function and symptoms like epistaxis	Loss of smell, nasal airway obstruction	CT scans can preoperatively suggest adenocarcinoma based on remodeling features
Yee et al. [26]	50 patients	Histopathological examination of nasal biopsy specimens	Changes in olfactory epithelium with sensorineural damage	Sensorineural damage	Pathology in nasal biopsy specimens reveals epithelial change and sensorineural damage in chronic rhinosinusitis
Stolovitzky et al. [15]	101 patients	Bioabsorbable implant for lateral wall insufficiency	Significant reduction in nasal obstruction over 6 months	Nasal obstruction	Stabilization of the lateral nasal wall improves symptoms
Kandathil et al. [14]	469 patients	Retrospective study comparing functional and cosmetic rhinoplasty patients	Lateral wall insufficiency is higher in patients with functional complaints	Nasal airway obstruction	Only patients with a positive modified Cottle maneuver should undergo repair
Sheen [12]	40 patients	Le Fort I osteotomy	Neurosensory deficit was a common finding among complications	Sinusitis	Proper surgical technique reduces complications
Lakshmi et al. [27]	31 patients	Infraorbital nerve paraesthesia recovery patterns post-surgery	Significant improvement noted after 3-6 months in the surgical group	Nerve paraesthesia	The infraorbital nerve’s palpebral branch shows maximum disruption, leading to paraesthesia

Discussion

Additional research has expanded the literature by exploring distinct conclusions. Kern [11] highlighted that inflammatory changes within the olfactory mucosa can lead to olfactory deficits. Damm et al. [9] demonstrated that nasal cavity volume can be modified through inferior turbinate resection and/or septoplasty. Nguyen et al. [23] revealed that obstructive sleep apnea impairs sensory detection at multiple upper-airway sites. Trotier et al. [24] emphasized the unique function of olfactory clefts, noting their distinct response to local inflammatory events. Georgel et al. [25] proposed that CT imaging can preoperatively identify adenocarcinomas in the olfactory cleft based on remodeling features. Yee et al. [26] underscored the diagnostic value of histopathological examination in understanding epithelial and sensorineural damage in chronic rhinosinusitis. Lastly, Lakshmi et al. [27] reported that the infraorbital nerve’s inferior palpebral branch is particularly susceptible to functional disruption, leading to paraesthesia in the infraorbital and malar regions. Collectively, these studies align with existing literature, reaffirming that sensory disturbances detrimentally affect patients’ quality of life and may exacerbate nasal airway obstruction [1-3].

A recurring theme among the findings is the necessity of understanding the anatomy, function, and pathophysiology of nasal abnormalities to optimize treatment strategies [7]. Consistent with André et al. [4] perspective, surgical interventions must be customized to address the specific site and nature of each disturbance, ultimately enhancing patients’ quality of life. The findings of this systematic review highlight the significance of sensory disturbances following lateral nasal wall surgery and their impact on patients’ quality of life. Our analysis of the 15 selected studies aligns with previous literature, emphasizing the importance of tailored surgical interventions to address these changes.

Recent studies provide additional insights and advancements. Research shows that sensory changes, such as olfactory dysfunction, occur in up to 45% of patients post-turbinate surgery [28]. Recent studies have provided valuable insights into the sensory changes experienced by patients following lateral nasal wall surgery, particularly concerning olfactory function. A systematic review focusing on inferior turbinate surgeries reported consistent improvements in olfactory function postoperatively, regardless of the surgical technique employed. This underscores the potential benefits of such interventions on sensory outcomes, despite not being the primary goal of this systematic review [29]. As limitations and future directions, we point out that the lack of a formal meta-analysis limits statistical robustness. Most studies also lack follow-up beyond 12 months, impacting the understanding of long-term sensory changes. Future research should include larger, randomized controlled trials with extended follow-up.

## Conclusions

The articles selected for this study are highly relevant, offering valuable insights that align with the research objectives. The findings from these studies consistently demonstrate a clear link between the sensory changes reported by patients post-surgery and the previously identified sensory disturbances. These disturbances include chronic rhinosinusitis, odor discrimination issues, poor sleep quality, inflammatory obstruction, damage to normal nasal tissue, loss of olfactory function, sensorineural impairment, sinusitis, nasal airway obstruction, severe allergic rhinitis, moderate to severe obstructive sleep apnea, and nerve paraesthesia.

The identification of these sensory disturbances across the reviewed articles strongly supports the conclusion that surgical interventions led to significant improvements in patients' quality of life. Furthermore, no major complications or adverse outcomes were reported in these studies. These findings underscore the necessity for surgeons to customize surgical techniques to address the specific sensory disturbances of each patient, ensuring the intervention is tailored to their individual needs for optimal outcomes. In conclusion, sensory disturbances are common but often temporary. Tailored surgical approaches and advanced techniques remain essential for optimizing patient outcomes and enhancing quality of life.
